# Fibroblast Growth Factor Receptor 3 Interacts with and Activates TGFβ-Activated Kinase 1 Tyrosine Phosphorylation and NFκB Signaling in Multiple Myeloma and Bladder Cancer

**DOI:** 10.1371/journal.pone.0086470

**Published:** 2014-01-23

**Authors:** Lisa Salazar, Tamara Kashiwada, Pavel Krejci, April N. Meyer, Malcolm Casale, Matthew Hallowell, William R. Wilcox, Daniel J. Donoghue, Leslie Michels Thompson

**Affiliations:** 1 Department of Psychiatry and Human Behavior, University of California Irvine, Irvine, California, United States of America; 2 Department of Biological Chemistry, University of California Irvine, Irvine, California, United States of America; 3 Medical Genetics Institute, Cedars-Sinai Medical Center, Los Angeles, California, United States of America; 4 Institute of Experimental Biology, Masaryk University and Department of Cytokinetics, Institute of Biophysics AS CR, v.v.i., Brno, Czech Republic; 5 Department of Pediatrics, UCLA School of Medicine, Los Angeles, California, United States of America; 6 Department of Chemistry and Biochemistry, University of California San Diego, La Jolla, California, United States of America; 7 Department of Neurobiology and Behavior, University of California Irvine, Irvine, California, United States of America; 8 Moores Cancer Center, University of California San Diego, La Jolla, California, United States of America; 9 Chao Family Comprehensive Cancer Center, University of California Irvine, Irvine, California, United States of America; Louisiana State University Health Sciences center, United States of America

## Abstract

Cancer is a major public health problem worldwide. In the United States alone, 1 in 4 deaths is due to cancer and for 2013 a total of 1,660,290 new cancer cases and 580,350 cancer-related deaths are projected. Comprehensive profiling of multiple cancer genomes has revealed a highly complex genetic landscape in which a large number of altered genes, varying from tumor to tumor, impact core biological pathways and processes. This has implications for therapeutic targeting of signaling networks in the development of treatments for specific cancers. The NFκB transcription factor is constitutively active in a number of hematologic and solid tumors, and many signaling pathways implicated in cancer are likely connected to NFκB activation. A critical mediator of NFκB activity is TGFβ-activated kinase 1 (TAK1). Here, we identify TAK1 as a novel interacting protein and target of fibroblast growth factor receptor 3 (FGFR3) tyrosine kinase activity. We further demonstrate that activating mutations in FGFR3 associated with both multiple myeloma and bladder cancer can modulate expression of genes that regulate NFκB signaling, and promote both NFκB transcriptional activity and cell adhesion in a manner dependent on TAK1 expression in both cancer cell types. Our findings suggest TAK1 as a potential therapeutic target for FGFR3-associated cancers, and other malignancies in which TAK1 contributes to constitutive NFκB activation.

## Introduction

Cancer is a complex disease arising from the acquisition of somatic mutations that dysregulate signaling pathways central to cell proliferation and survival, angiogenesis, and metastasis. Dysregulation of FGFR3 signaling has been implicated in several cancer types, most notably urothelial cell carcinoma (UC) and multiple myeloma (MM). Urothelial cell carcinomas account for more than 90% of bladder cancers, which have a worldwide incidence of over 350,000 new annual diagnoses and rank as the third most common malignancy in men and the tenth most common in women in the United States [1]. Overexpression or activating mutation of FGFR3 is the most frequent genetic alteration in UC (Reviewed in [2]). Multiple Myeloma, a cancer of terminally differentiated B cells, is the second most common hematologic cancer with an American Cancer Society estimate of 22,350 new cases for 2013. Among the cases of MM with the poorest prognosis are those 15% with the t(4;14) translocation, which targets both FGFR3 and MMSET (Reviewed in [3]–[5]). Recent studies indicate that this translocation may be the major clone at diagnosis or, conversely, observed only at the time of relapse [6]. However, the mechanism underlying the aggressiveness of t(4;14) myeloma remains unclear and the relative contribution of FGFR3 and MMSET as putative oncogenes is controversial, as 25% of t(4;14) tumors lack FGFR3 expression. The acquisition of FGFR3-activating mutations (5–10% of t(4;14) cases) with disease progression indicates a role for FGFR3 in MM pathogenesis, and early studies demonstrate the oncogenic potential of activated mutant FGFR3 [4]. It was also more recently demonstrated that wild-type FGFR3, as is expressed in most FGFR3-positive t(4;14) tumors, can contribute to B cell oncogenesis [7]. Furthermore, a wealth of preclinical data demonstrate the effectiveness of receptor tyrosine kinase inhibitors and neutralizing antibody against MM cells expressing FGFR3-activating mutations and wild-type receptor (reviewed in [3]–[5]). Similarly, inhibition of FGFR3 can induce cell cycle arrest and/or apoptosis in UC [8], [9] both *in vitro* and *in vivo*, providing validation that FGFR3 and downstream signaling pathways represent potentially relevant therapeutic targets for the treatment of FGFR3-associated cancers.

FGFR3 is one of four tyrosine kinase receptors that mediate the effects of FGFs on diverse cellular processes, including proliferation, differentiation, and migration. Receptor activation triggers signal transduction pathways implicated in oncogenesis, including the Ras/ERK/MAPK, PLCγ/PKC, PI3K, and STAT pathways [10]. More recent evidence indicates that FGF receptor signaling can also activate NFκB [11], [12], the aberrant activation of which is frequently observed in human cancer [13], [14] and closely correlates with cancer hallmarks [15]. A key intermediate in NFκB signaling, TGFβ-activated kinase 1 (TAK1), functions downstream of multiple signaling pathways, regulating cell survival, differentiation, and inflammatory responses [16], and stands as a key IKK-kinase of the canonical NFκB pathway [17]. Chemical and genetic inhibition of TAK1 promotes apoptosis in skin tumors [18] and a subset of colon cancers [19], as well as decreasing chemoresistance in breast and colon cancer cells [20] and chemoresistance and NFκB activity in pancreatic cancer cells in culture [21]. Furthermore, suppression of TAK1 signaling reduces NFκB activation in human head and neck squamous cell carcinoma cell lines [22], ovarian carcinoma cells [23], and breast cancer cell lines [24], and blocks breast cancer cell adhesion, invasion, and metastasis in vitro [25]. TAK1 has not been investigated in the context of MM or bladder cancer; however, it’s downstream target, NFκB, has emerged as one of the most potent drivers of tumorigenesis in MM, with as many as 82% of MM samples expressing signature activation molecules [26], [27]. Consistent with this key oncogenic role, several drugs that are effective against MM, including bortezomib, thalidomide, and lenalidomide, block activation of NFκB (reviewed in [28]). In UC, suppression of NFκB activity potentiates the apoptotic effects of chemotherapeutic agents and cytokines [29], [30].

Using a combination of yeast two-hybrid and microarray genetic screening coupled with systems pathway analysis, we identify TAK1 as a novel interactor and target of FGFR3 tyrosine kinase activity. We further demonstrate a role for TAK1 as a positive regulator of NFκB activity downstream of FGFR3 in both multiple myeloma and urothelial cell carcinoma, two cancers with demonstrated FGFR3 involvement [10], [31], with modulatory effects on cell adhesion.

## Methods

### Cell Culture and Transfection

FGFR3-negative (RPMI-8226) and wild-type (LP1) human MM cell lines were obtained from the German Collection of Microorganisms and Cell Cultures [DSMZ; Braunschweig, Germany]; FGFR3 mutant MM cells (KMS-11; Y373C) derived from a MM patient and established at Kawasaki Medical School [32], were generously provided by Dr. P Leif Bergsagel. The mutant FGFR3 bladder cancer cell line, MGHU3 (Y375C), a kind gift from Dr. Margaret Knowles (University of Leeds, Leeds, UK), was derived from a grade 1 tumor [33]. MM and UC cells were maintained in RPMI 1640 (Hyclone; Thermo Scientific, Rockford, IL) and HeLa and HEK293 cells (ATCC) in DMEM (Hyclone), both media supplemented with 10% fetal bovine serum (Invitrogen). Transient transfection of HeLa and HEK293 cells was achieved using Lipofectamine 2000 (Life Technologies; Grand Island, NY) according to the manufacturer’s protocol and MM and UC transfected lines using the Neon system (Life Technologies). Following the manufacturer’s procedure, 1×10^6^ UC or 2×10^6^ MM cells were suspended in 100 µl suspension solution containing 5 µg siRNA (Dharmacon) or plasmid and pulsed under program 3 for UC and program 15 (KMS-11) or 20 (RPMI-8226) for MM cells.

### Antibodies and Reagents

FGFR3 antibody (B-9, C-15) and FGFR1/2/4 antibodies were from Santa Cruz Biotechnology, Inc. (Santa Cruz, CA). Antibodies to TAK1, ERK, phospho-ERK, phospho-Tyrosine (4G10), p65 and p84 were from Millipore (Billerica, MA), as was normal rabbit IgG. Recombinant human FGF1 was obtained from R&D Systems (Minneapolis, MN) and PD173074 from Sigma (St. Louis, MO). Non-targeting and TAK1-specific siRNA (both ON-TARGETplus SMART-pool) were purchased from Dharmacon (Thermo Scientific). Human Collagen type IV was from Sigma.

### Plasmid Constructs

Untagged or C-terminally FLAG-tagged FGFR3 constructs have been previously described [34], as were constructs for FGFR2, and −4 [35], [36]. The vector expressing FGFR1 was generated by cloning full-length human FGFR1 ORF into the pcDNA3.1 vector (Life Technologies), according to the manufacturer’s protocol. HA-tagged murine TAK1 was kindly provided by Dr. Hiroaki Sakurai (University of Toyama, Toyama, Japan). NF-κB-Luc was from Agilent Technologies (Santa Clara, CA), and pRL-TK control *Renilla* from Promega (Madison, WI).

### Yeast 2-hybrid

A yeast two-hybrid screen was performed as previously described [37]. Briefly, wild-type or constitutively active (K650E) sequences of the human FGFR3 cytoplasmic domain amino acids 399–806) were fused to the LexA DNA-binding domain in the pBTM116 plasmid and used to screen a human chondrocyte library encoding fusion proteins with the Gal4 activation domain (BD Biosciences Clontech, Palo Alto, CA) in the L40 strain of *Saccharomyces cerevisiae*. Transformants were grown 3–4 days on selective media and the resulting colonies subjected to a β-galactosidase filter lift assay. Subsequent domain-mapping was performed similarly, using truncated FGFR3 cytoplasmic domain sequences as bait, paired with full-length or C-terminal TAK1 sequences as prey.

### Immunoprecipitation and Immunoblot Analysis

**Figure 1:**
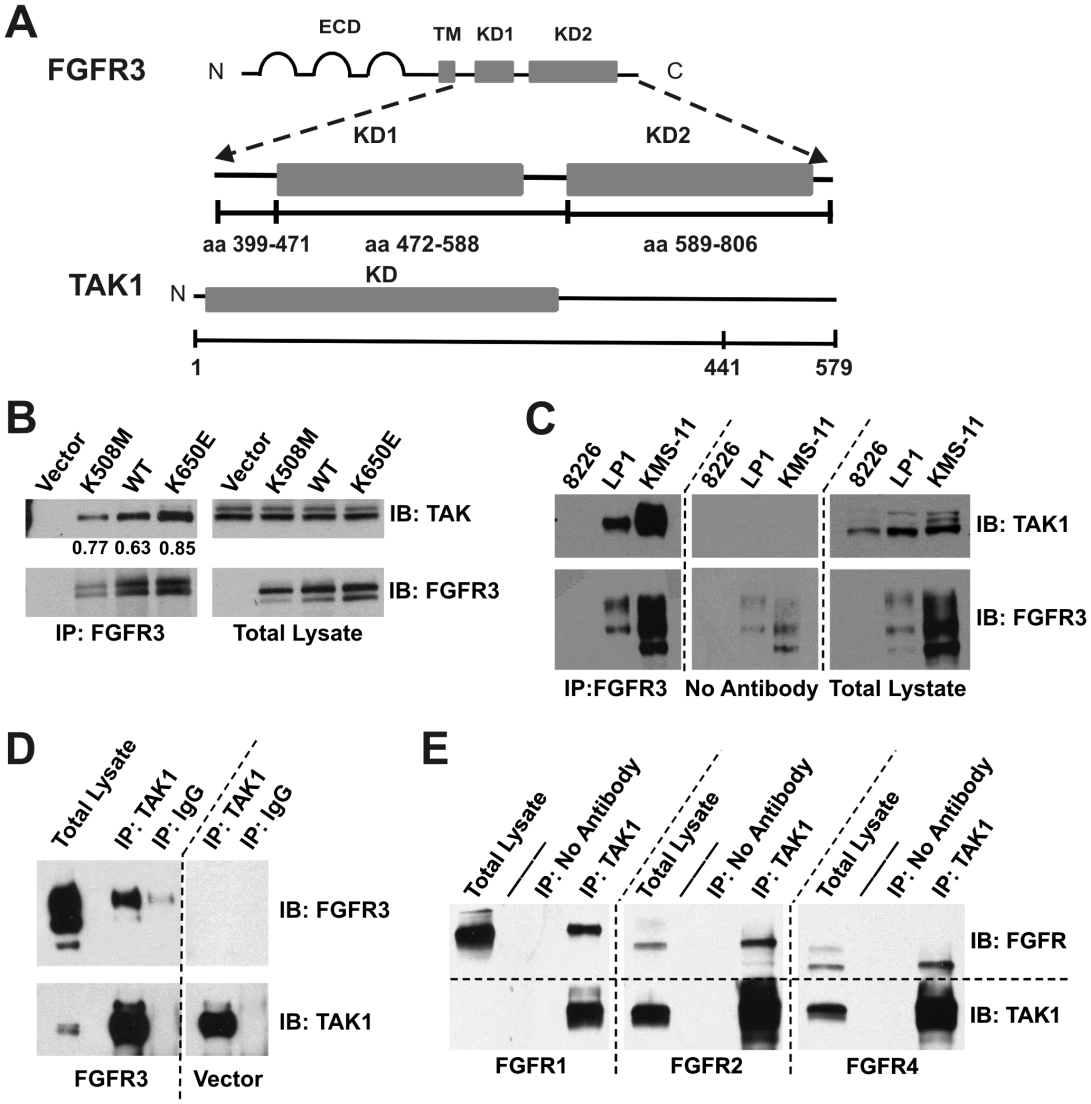
TAK1 interacts with FGFR3. (**A**) Schematic of FGFR3 and TAK1 domains used for yeast two-hybrid screening and subsequent mapping of the interaction in yeast. (**B**) Endogenous TAK1 interacts with kinase-dead (K508M), wild-type, and constitutively active (K650E) FGFR3 in HeLa cells. Numerical values represent the ratio of TAK1 co-precipitated with FGFR3. (**C**) FGFR3 and TAK1 (both endogenous) interact in LP1 (FGFR3WT) and KMS-11 (FGFR3Y373C) multiple myeloma cell lines. The 8226 line is negative for FGFR3. (**D**) Endogenous TAK1 interacts with FGFR3 in MGHU3 bladder cancer cells transfected with wild-type FGFR3. MGHU3 also express the FGFR3 activating mutation, Y375C. (**E**) TAK1 (endogenous) interacts with overexpressed FGFR1, −2, and −4 in HEK293 cells. TAK1 in the FGFR1-transfected total lysate is detectable upon longer exposure (data not shown). For all blots (**B-E**), immunoprecipitations were performed from 1mg total lysate using the antibody indicated. Blots were first probed for the interaction partner being tested, then stripped and re-probed for the immunoprecipitated protein. 20 µg total lysate was similarly probed to control for expression and loading. Arrow indicates TAK1. Multiple FGFR3 bands represent various glycosylation intermediates and appear as previously published [45], [85]. Four independent experiments were performed for each panel.

Cells were washed in cold PBS containing 1% sodium orthovanadate and lysed in 1% Nonidet P-40 lysis buffer (20 mMTris-HCl, pH7.5, 137 mM NaCl, 1% Nonidet P-40, 5 mM EDTA, 50 mM NaF, 1 mM sodium orthovanadate, 1 mM phenylmethylsulfonyl fluoride, 10 µg/ml aprotinin). Lysates were pre-cleared with protein A-Sepharose beads (Millipore) and immunoprecipitations performed overnight with 2 µg antibody. Immunoprecipitates were washed 3 times with lysis buffer, boiled 5 min in sample buffer and resolved by 10% SDS-PAGE. Membranes were blocked with Starting Block blocking buffer (Thermo Scientific) and probed as indicated. Antibody binding was detected using SuperSignal West Pico or SuperSignal West Dura chemiluminescent substrate (Thermo Scientific). To reprobe with other antibodies, membranes were stripped of bound antibodies using Restore stripping buffer (Thermo Scientific). Where indicated, densitometry was performed using ImageJ. It should be noted that co-immunoprecipitations from Figure 1E were performed using 30 µl washed Dynabeads (Life Technologies) instead of protein A-sepharose beads and without a preclear step, but were otherwise treated as described above.

### Mass Spectrometry Analysis

HEK293 cells were transfected with expression plasmids for TAK1 and constitutively active (K650E) FGFR3. After 24 hours, cell lysates were prepared as described [38], [39]. TAK1 immune complexes were precipitated with anti-TAK1 antibody at 4°C overnight, collected with Protein A-sepharose for an additional 2 hours, and then digested with trypsin. Peptides were analyzed by the Proteomics Facility of the Sanford-Burnham Medical Research Institute using immobilized metal affinity chromatography/nano-liquid chromatography/electrospray ionization mass spectrometry (IMAC/nano-LC/ESI-MS) [38], [39].

### FGFR3 *In-vitro* Kinase Assay

The FGFR3 kinase assays were carried out as previously described [40]. Briefly, kinase reactions were performed in 50 µl of kinase buffer (60 mMHepes-NaOH pH 7.5, 3 mM MgCl_2_, 3 mM MnCl_2_, 3 µM Na_3_VO_4_, 1.2 mM DTT) supplemented with 2.5 µg PEG, 100 µM ATP and recombinant human TAK1 (500 ng; Abnova, Taipei City, Taiwan) as a substrate. The recombinant active FGFR3 intracellular domain (397-End; SignalChem, Richmond, CA) was used at 300 ng per reaction.

### Microarray Procedures and Analysis

Cells were transfected with 5 µg non-targeting or TAK1-specific siRNA and allowed to recover overnight. The next day, cells remained untreated or received 100 nM PD173074 for 48 hr before RNA isolation. Each treatment was prepared as triplicate samples. Total RNA was processed as recommended by Affymetrix, Inc. Briefly, RNA was isolated using TRIzol (Life Technologies) and passed through RNeasy spin columns (Qiagen, Valencia, CA) for further clean up. The UCI DNA Microarray Core Facility then quantified total RNA by NanoDrop (Thermo Scientific) and tested for purity using the Agilent Bioanalyzer 2100 (Agilent Technologies). The Ambion WT expression kit (Life Technologies) was used to prepare RNA samples for whole transcriptome microarray analysis. Two ug of the labeled, fragmented single-stranded cDNA was then hybridized to probe sets on a Human AffymetrixGeneChip 1.0ST array. Arrays were scanned using the GeneChip Scanner 3000 7 G and Command Console Software v. 3.2.3. Results are available through the Gene Expression Omnibus (GEO) repository (accession number GSE52452).

Data were imported into Partek Genomics Suite Version 6.6 software with the following operations being done to prepare the data for statistical analysis: 1) RMA Background Correction, 2) Quantile Normalization, 3) Log base 2 transformation, and 4) Summary of Probe sets using mean value. Statistical analysis consisted of one-way ANOVA with a single categorical variable, and gene lists were generated for those genes with fold-change magnitude >2 and p-value with a false discovery rate (FDR) <.05.

Gene lists were then imported into Ingenuity Systems Pathway Analysis (IPA) software, which has functions for generating gene networks, sorting genes into various functional and other categories, and for overlaying genes onto known signaling pathways, coloring by fold change or some other value.

### Quantitative RT-PCR

Total RNA was isolated from MGHU3 and KMS-11 cells using TRIzol (Life Technologies) and passed through RNeasy spin columns (Qiagen) for further clean up. Random-primed cDNA synthesis was performed on 1 µg total RNA using the Superscript III RT Kit (Life Technologies). All primer pairs were intron-spanning and a no RT control was included. Primer pairs were as follows: Actin reverse AGGTGTGGTGCCAGATTTTC and forward GGCATGGGTCAGAAGGATT, GAPDH reverse GCCAGTGGACTCCACGAC and forward CAACTACATGGTTTACATG, DFNA5 *reverse* CAGGTTCAGCTTGACCTTCC and *forward* ACCAATTTCCGAGTCCAGTG, GSTA1 reverse CCGTGCATTGAAGTAGTGGA and forward ACGGTGACAGCGTTTAACAA, PSCA reverse GTTCTTCTTGCCCACGTAGT and forward CAGGTGAGCAACGAGGAC, BAMBI reverse GAAGTCAGCTCCTGCACCTT and forward TGCACGATGTTCTCTCTCCT, TNFAIP3 reverse CGCTGTTTTCCTGCCATTTC and forward GATAGAAATCCCCGTCCAAGG, SGK1 reverse TGTCAGCAGTCTGGAAAGAGAAGT and forward CGGAATGTTCTGTTGAAGAATGTG.

### NFκB Luciferase Assay

Cells were transfected with 5 µg non-targeting or TAK1-specific siRNAs. Twenty-four hours later, cells were transfected with NF-κB-Luc and pRL-TK control *Renilla* at a ratio of 3∶1 and were allowed to recover for 24 hours. Where indicated, cells were simultaneously transfected with the indicated FGFR3 plasmids. Cells were then serum starved overnight, followed by an 8 hour treatment with 40 ng/ml FGF1. Luciferase activity was detected using a dual-luciferase reporter assay (Promega: Madison, WI). Differences in NFκB activity following TAK1 silencing under each treatment condition were statistically analyzed using an unpaired two-tailed t-test.

### Cell Fractionation

MGHU3 cells were transfected with 5 µg non-targeting or TAK1-specific siRNAs. Forty-eight hours later, cells were serum starved overnight, then treated with 40 ng/ml FGF1 for 0, 5 or 60 minutes. Cells were collected then fractionated, using a protocol adapted from [41].

### Cell Adhesion Assay

Cells were transfected with 5 µg non-targeting or TAK1-specific siRNA and allowed to recover overnight. The next day, cells remained untreated or received 50 nM PD173074 for 48 hours before plating of the adhesion assay. Cells treated with FGF1 were serum-starved overnight and treated with ligand 1 hour prior to plating and throughout the duration of the assay. Adhesion assays were performed 72 hours post-transfection. Briefly, ninety-six-well plates were coated overnight at 4°C with 1 µg/ml collagen IV, blocked with 1% BSA for 1 hour at 37°C, and washed twice with PBS and once with serum-free medium. Cells were collected and seeded at 5×10^4^ on the pre-coated plates, in the presence of the treatment indicated. Cells were allowed to adhere 3 hours at 37°C, wells were washed 3 times with PBS to remove non-adherent cells, and adherence determined following 4 hour incubation with Calcein-AM (Life Technologies) by measuring fluorescence intensity at Ex/Em 490/520 nm. Statistical analysis of differences in cellular adhesion following TAK1 silencing under each treatment condition was performed using an unpaired two-tailed t-test.

## Results

### FGFR3 Interacts with TAK1

The identification of protein interactions can provide critical information about specific signaling pathways and identify novel potential therapeutic targets. In MM, the specific role of ectopically expressed FGFR3 in a subset of cases remains controversial, while in bladder cancer, FGFR3 has been recently implicated as an important driver of proliferation [42]. We took a systematic approach to gaining a better understanding of FGFR3 signaling in associated cancers through the identification of new FGFR3 protein interactions using a yeast two hybrid (Y2H) assay. The cytoplasmic domain of human FGFR3 (amino acids 399–806), containing the wild-type sequence or the strongly activating K650E mutation, was used as bait to screen a primary human chondrocyte cDNA library (Fig. 1A) as described [37]. This library was chosen as FGFR3 is highly expressed in chondrocytes, and the strongly activating K650E mutation, present in a subset of both MM and UC [43], is present in the intracellular tyrosine kinase domain. Potential interactions were identified by the filter lift β-galactosidase assay, sequenced, and re-tested for interaction in yeast. Among the interactions identified were signal transduction proteins, including the p85 regulatory subunit of PI3-kinase [37], and TAK1. The yeast prey plasmid consisted of the C-terminal 138 amino acids of TAK1 (amino acids 441–579) indicating that this region of TAK1 is involved in binding FGFR3. We further determined by Y2H using FGFR3 domain constructs (Fig. 1A) that the region encompassing the second half of the tyrosine kinase domain of FGFR3, containing the activation loop of the receptor and C-terminal tail of FGFR3 (amino acids 589–806), is sufficient for the FGFR3-TAK1 interaction. To confirm the FGFR3-TAK1 interaction in mammalian cells and, specifically, FGFR3-associated cancer cell lines, human FGFR3 constructs, including wild-type, kinase-dead and constitutively active (K650E) sequences, were transiently expressed in HeLa cells. TAK1 co-immunoprecipitated with all FGFR3 sequences tested; demonstrating that FGFR3 and TAK1 interact in mammalian cells and that receptor activation is not required (Figure 1B). Endogenous FGFR3 in two t(4;14) MM cell lines, LP-1 (wt) and KMS-11 (Y373C) [44], [45], also interacted with TAK1 by co-immunoprecipitation (Figure 1C). As we observed FGFR3 levels were considerably lower in MGHU3 than MM lines, FGFR3^WT^ was overexpressed for adequate detection of a TAK1-FGFR3 interaction in MGHU3 (Figure 1D). Note that the MGHU3 UC cells carry the same FGFR3 mutation as the KMS-11 MM cells. An FGFR3-TAK1 interaction was also evaluated in RT-112 UC cells, which express a truncated wild-type FGFR3 (amino acids 1–758) in fusion with transforming acid coiled coil 3 (TACC3) sequences (residues 433–838) [46]. We were unable to convincingly detect the interaction in this line, further supporting the importance of FGFR3 C-terminal sequences in the interaction with TAK1 (data not shown). Finally, we also utilized mass spectrometry to characterize proteins recovered in TAK1 immunoprecipitates. Following expression of both activated FGFR3-K650E and TAK1 in HEK293 cells, TAK1 immunoprecipitates were analyzed by immobilized metal affinity chromatography/nano-liquid chromatography/electrospray ionization mass spectrometry (IMAC/nano-LC/ESI-MS) [38], [39]. In three independent samples, in addition to significant coverage of TAKI as expected, FGFR3-derived peptides representing 48% coverage overall were unambiguously identified, as presented in Table 1. Collectively, these results provide evidence of a novel interaction between FGFR3 and TAK1 that does not require activation of the receptor. There is precedence for this with the FRS2 adaptor, which interacts with FGF receptors constitutively, yet only activates downstream signaling (ERK/MAPK) upon receptor activation [47]. We further demonstrate that FGFR1, 2 and 4 transiently expressed in HEK293 cells, also interact with TAK1 (Figure 1E), suggesting that the interaction is broadly relevant to FGF receptor signaling.

**Table 1:** Mass spec analysis identifies FGFR3 as binding partner of TAK1.

Experiment	TotalIndependentSpectra	Peptide Sequence	NSP Adjusted Probability	TotalInstances	AAResidues
A	360	VAIVAGASSESLGTEQR	0.9998	1	014–030
A	360	IVAGASSESLGTEQR	0.9998	5	016–030
A	360	DGTGLVPSER	0.9999	8	077–086
A	360	VLVGPQR	0.9979	2	087–093
A,C	1460	LQVLNASHEDSGAYSCR	0.9999	69	094–110
A	360	VLCHFSVR	0.9999	10	117–124
A,C	580	VTDAPSSGDDEDGEDEAEDTGVDTGAPYWTRPER	1	26	125–154
A	360	KLLAVPAANTVR	1	4	162–173
A,C	580	LLAVPAANTVR	0.9906	20	163–173
A	360	FRCPAAGNPTPSISWLK	0.9999	2	174–190
A,B,C	677	CPAAGNPTPSISWLK	1	25	176–190
A	360	HQQWSLVMESVVPSDR	1	7	208–223
A	360	GNYTCVVENK	0.9999	1	224–233
A,B,C	677	QTYTLDVLER	1	51	239–248
A	360	HVEVNGSKVGPDGTPYVTVLK	1	2	290–310
A,B,C	897	VGPDGTPYVTVLK	0.9999	56	298–310
B,C	1611	RQVSLESNASMSSNTPLVR	0.9989	21	421–439
A,B,C	2262	QVSLESNASMSSNTPLVR	0.9999	146	422–439
C	220	ASMSSNTPLVR	0.9008	3	429–439
B,C	1611	IARLSSGEGPTLANVSELELPADPK	0.9999	20	440–464
C	440	IARLSSGEGPTLANVSELELPADPKWELSR	0.9999	4	440–469
A,B,C	1628	LSSGEGPTLANVSELELPADPK	0.9994	46	442–464
B,C	220	LSSGEGPTLANVSELELPADPKWELSR	0.9999	7	442–469
B	97	LTLGKPLGEGCFGQVVMAEAIGIDKDR	0.999	1	472–498
A,C	580	AAKPVTVAVK	0.998	3	499–508
A	360	MLKDDATDKDLSDLVSEMEMMK	0.9992	2	509–530
A,C	800	RPPGLDYSFDTCKPPEEQLTFK	0.997	15	571–592
A,B,C	897	DLVSCAYQVAR	0.9999	35	593–603
A,C	360	GMEYLASQK	1	6	604–612
A,B,C	994	NVLVTEDNVMK	0.9999	60	622–632
A	360	IADFGLAR	1	10	633–640
A,C	800	DVHNLDYYK	0.9999	11	641–649
A	360	WMAPEALFDR	1	10	660–669
A	360	MDKPANCTHDLYMIMR	1	2	713–728
A	360	ECWHAAPSQRPTFK	1	12	729–742
A,C	580	QLVEDLDR	0.9982	5	743–750

Mass spectrometry analysis of TAK1 immune complexes prepared from HEK293 cells identifies FGFR3 as a binding partner. The table shows recovered FGFR3 peptides (IPI Protein Index Identifier: IPI00027174,IPI00220253). Amino acid residues refer to the standard FGFR3 protein P22607 (FGFR3_HUMAN) UniProtKB/Swiss-ProtGenBan, 806 aa total length. NSP refers to “number of sibling peptides.”

### FGFR3 can Tyrosine Phosphorylate TAK1 *in vitro*

**Figure 2:**
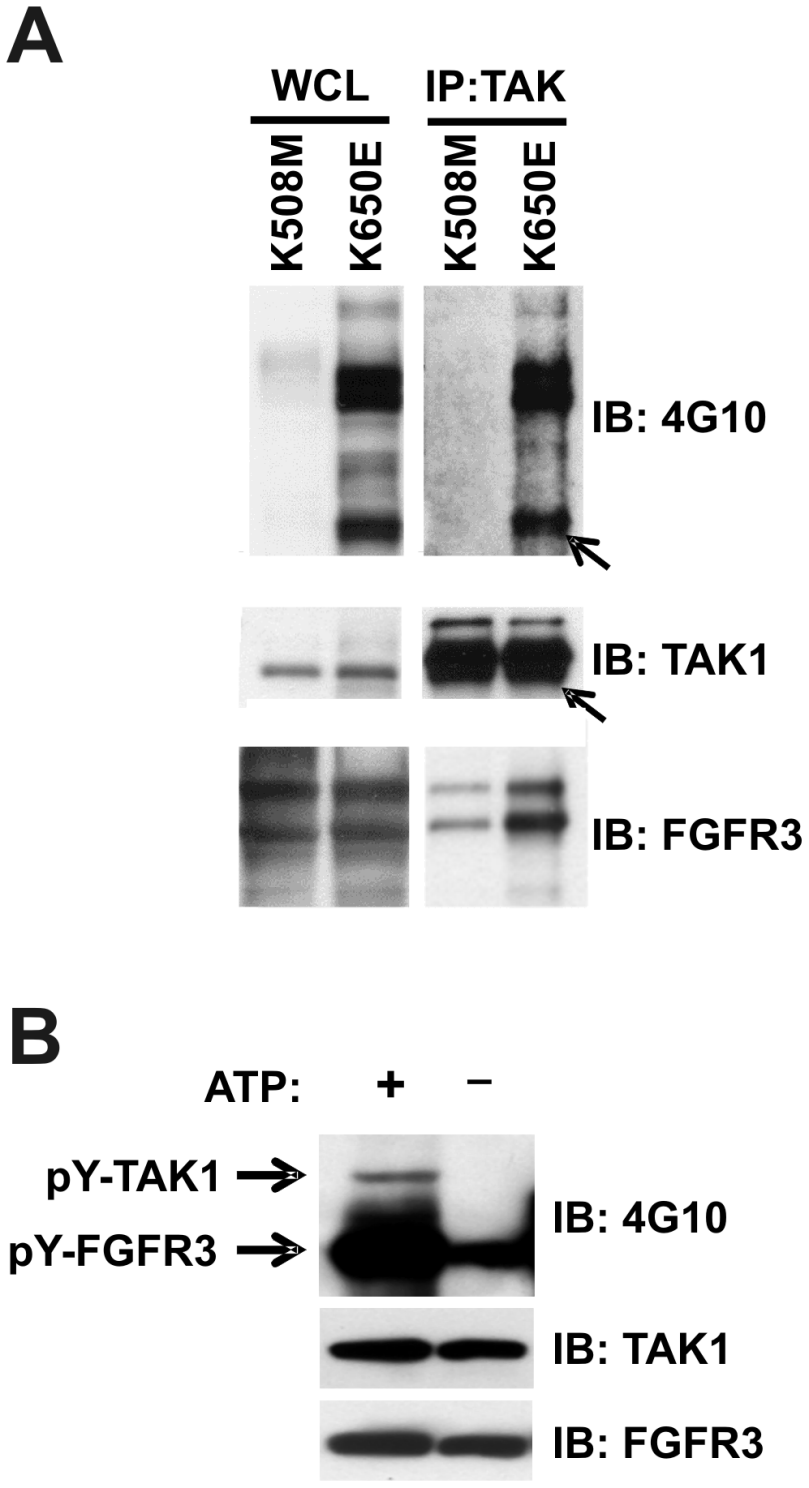
FGFR3 can tyrosine phosphorylate TAK1. (**A**) HEK293 cells transfected with FGFR3^K508M^ or FGFR3^K650E^. Twenty-four hours following transfection, cells were lysed and TAK1 immunoprecipitated from 1 mg total lysate. Immunoprecipitates were resolved by SDS-PAGE, blotted, and probed with 4G10 antibody. Arrow indicates TAK1. Representative of six experiments. (**B**) A cell-free kinase assay was performed using recombinant human TAK1 has a substrate for recombinant human FGFR3 (tyrosine kinase domain). Tyrosine phosphorylation was visualized by immunoblotting with 4G10 antibody. Representative of four experiments.

TAK1 activation requires Ser/Thr phosphorylation at multiple residues in the activation loop (reviewed in [48], [49]). Although tyrosine phosphorylation of TAK1 has not been previously reported, FGFR3 functions as a tyrosine kinase; therefore, we evaluated the possibility that FGFR3 might tyrosine phosphorylate TAK1. Indeed, we found that TAK1 was tyrosine phosphorylated in HEK293 cells transiently expressing constitutively active FGFR3 (K650E), but not the kinase-dead receptor (K508M), indicating that activated FGFR3 can either directly or indirectly tyrosine phosphorylate TAK1 (Figure 2A). TAK1 tyrosine phosphorylation was further observed in a cell-free kinase assay using recombinant TAK1 and the kinase-active intracellular domain of FGFR3, indicating that TAK1 can be a direct target of FGFR3 tyrosine kinase activity (Figure 2B).

### Gene Expression Analysis Identifies NFκB as a Signaling Hub for FGFR3 and TAK1 Integration

TAK1 is a key mediator of signaling cascades leading to activation of the NFκB and AP-1 transcription factors, which each modulate expression of genes involved in oncogenesis and apoptosis (Reviewed in [16], [50]). To begin to investigate the integration of TAK1 and FGFR3 signaling in cancer cells, we performed a comparative microarray analysis of gene expression in the MGHU3 bladder cancer cell line, which expresses the FGFR3 Y375C activating mutation and exhibits strong responses to the FGF receptor-specific PD173074 inhibitor as assessed by ERK phosphorylation [9]. To identify genes that are dependent on both FGFR3 and TAK1 signals, MGHU3 cells were transfected with non-targeting or TAK1-specific siRNA, and each subset further treated with PD173074, or vehicle control. One way ANOVA with fold change magnitude >2 and p-value with FDR <.05 was used to generate gene lists. TAK1 siRNA versus non-targeting siRNA samples yielded 39 gene changes reflecting TAK1 specific genes in the presence of FGFR3 signaling. TAK1 siRNA plus PD173074 versus non-targeting siRNA plus PD173074 samples yielded 105 gene changes reflecting TAK1 specific genes in the absence of FGFR3 signaling. To discern changes that are dependent on both FGFR3 and TAK1, genes that show statistically significant gene changes arising from TAK1 knockdown only in the presence of FGFR3 signaling but not in its absence were selected. Overlapping genes from the set of 105 TAK1 gene changes in the absence of FGFR3 signaling were removed from the 39 TAK1 gene changes in the presence of FGFR3 activity. The 13 unique genes that remained as significantly altered in these conditions represent genes that reflect both TAK1 and FGFR3 signaling (Table 2).

**Table 2:** FGFR3 and TAK1 alter gene expression in Bladder Cancer cells.

Gene Symbol	Gene Name	Accession Number	p-value	Direction
ACSL1	acyl-CoA synthetase long-chain family member 1	NM_001995	7.30E-08	Down
VGLL1	vestigial like 1	NM_016267	5.70E-07	Down
ARRB1	arrestin, beta 1	NM_004041	4.82E-07	Down
SCNN1G	sodium channel, non-voltage-gated 1, gamma subunit	NM_001039	1.93E-07	Down
MT2A	metallothionein 2A	NM_005953	1.27E-07	Up
SGK1*	serum/glucocorticoid regulated kinase 1	NM_001143676	5.65E-06	Down
PSCA*	prostate stem cell antigen	NM_005672	2.28E-06	Down
BAMBI*	BMP and activin membrane-bound inhibitor homolog	NM_012342	3.47E-05	Down
TNFAIP3*	tumor necrosis factor, alpha-induced protein 3	NM_006290	1.55E-05	Down
TRIM31	tripartite motif containing 31	NM_007028	1.28E-05	Down
DFNA5*	deafness, autosomal dominant 5	NM_004403	5.18E-04	Up
GSTA1*	glutathione S-transferase alpha 1	NM_145740	1.29E-04	Down
MGAT4A	mannosyl (alpha-1,3-)-glycoprotein beta-1,4-N-acetylglucosaminyltransferase,isozyme A	NM_012214	1.11E-04	Down

A microarray experiment was performed using MGHU3 bladder cancer cells transfected with control or TAK1 siRNA, then treated with or without FGFR inhibitor, PD173074. One way ANOVA with fold change magnitude >2 and a p-value with FDR <0.05 was used to generate gene lists. Lists compared samples transfected with control verses TAK1 siRNA and samples transfected with control versus TAK1 siRNA that were additionally treated with PD173074. Genes common to both comparisons were then removed from the control versus TAK1 siRNA list, and are reflected in the table above. Asterisks indicate further validation done by qPCR in separate experiments using MGHU3 cells or KMS11 MM cells treated as in the original microarray experiment. For TNFAIP3, validation in MM cells was dependent on ligand presence. Expression of GSTA1 in MM cells was not detectable.

**Figure 3:**
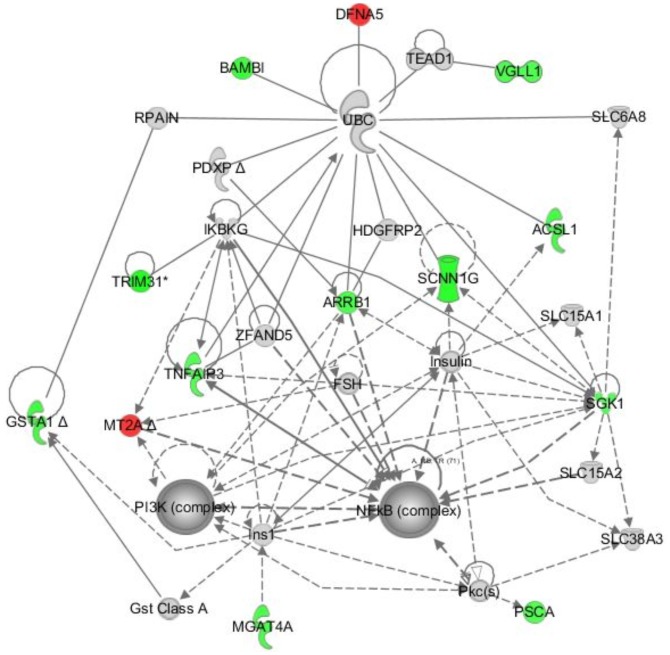
Genes dependent on FGFR3 and TAK1 signaling map to signaling networks with a major hub around NFκB. The 13 unique FGFR3 and TAK1 dependent genes (Table 1) were evaluated by Ingenuity Pathway Analysis (IPA) software, producing a single network (network score of 40) containing major hubs at NFκB and PI3K. IPA molecular shapes include: complex/group (NFκB, PI3K), cytokine/growth factor (IKBKG, SGK1), enzyme (ACSL1, GSTA1, MGAT4A, PDXP, TNFAIP3, UBC), ion channel (SCNNIG), transcriptional regulator (TEAD1, VGLL1), transporter (SLC6A8, SLC15A1, SLC15A2, SLC38A3), and other (ARRB1, BAMBI, DFNA5, FSH, GST Class A, HDGFRP2, Ins1, Insulin, MT2A, PKC(s), PSCA, RPAIN, TRIM31, ZFAND5). IPA Relationships: solid lines indicate direct interaction; dashed lines indicate indirect interaction; filled arrows indicate “acts on”; open arrows indicate “translocates to”; –| indicates “inhibits” and –|▸ indicates “acts on and inhibits. Green/red indicates genes down/up-regulated in the microarray (Table 1).

We chose 6 genes from the list of 13 for validation based on their relevance in cancer, and found that the observed changes were reproducible by qPCR, both in MGHU3 and the KMS-11 MM line treated with TAK1 knockdown and/or FGF receptor inhibition as described above for the microarray analysis (Table 2). The only exception is GSTA1, which has very low levels of expression in MM cells. Finally, input of the list of the 13 genes into Ingenuity Systems Pathway Analysis Tool (IPA) resulted in a single gene network (network score 40) with a major hub around NFκB (Figure 3). These results suggest a critical intersection between FGFR3 and TAK1 signaling that may impact NFκB activation and thus cancer pathogenesis in FGFR3-associated cancers. A second hub focused around PI3K is consistent with our previous results showing an interaction between FGFR3 and the p85 regulatory subunit of PI3K [37].

### Activated FGFR3 Positively Regulates NFκB Activity through TAK1

**Figure 4:**
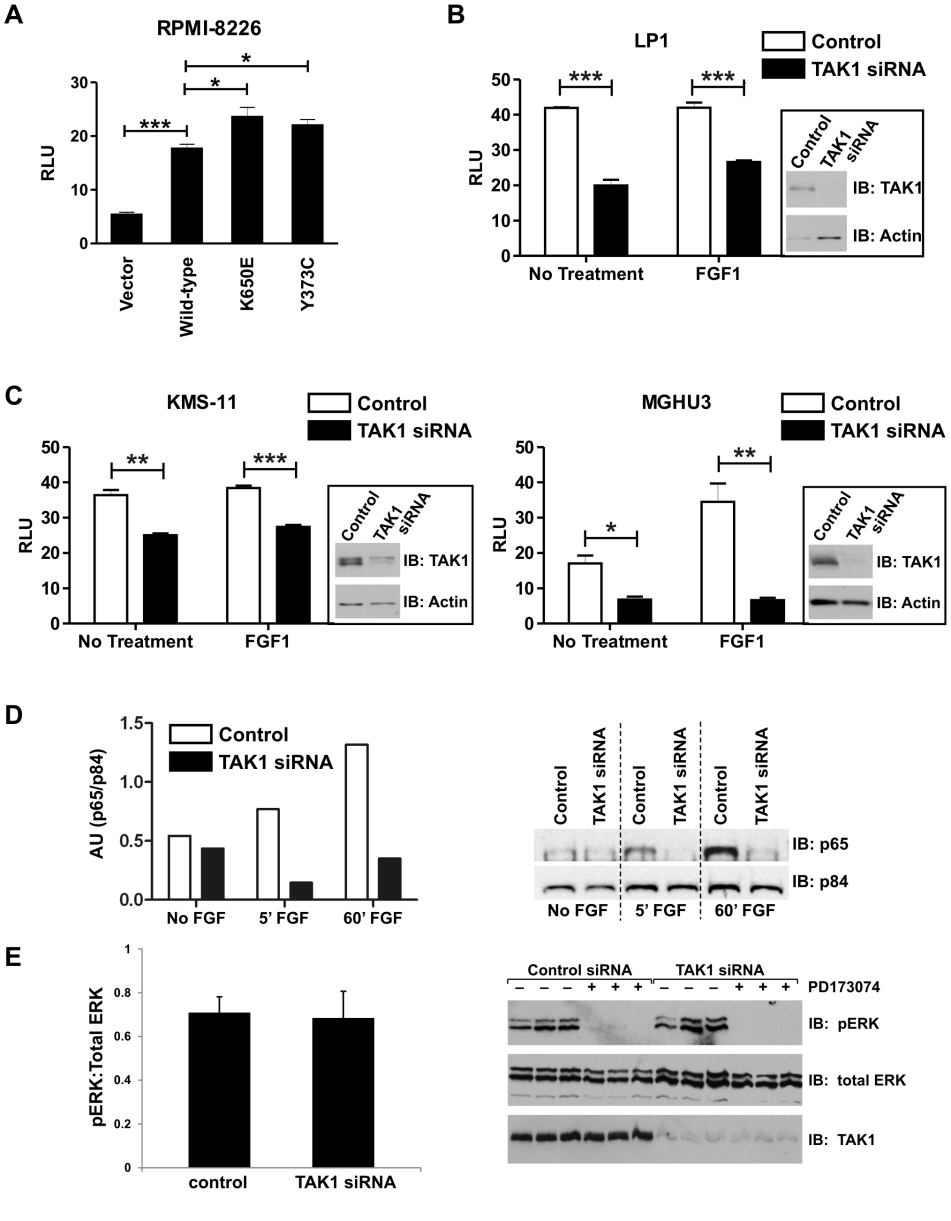
TAK1 knockdown inhibits FGFR3-dependent NFκB activation. (**A**) 8226 (FGFR3 negative) MM cells were transfected with 5 µg FGFR3 constructs or empty vector, and NF-κB-Luc and pRL-TK control *Renilla* reporter at a ratio of 3∶1, respectively for 48 hours. Cells were then lysed and assayed for dual-luciferase activity. (**B, C**) FGFR3-expressing bladder and MM cell lines were transfected with control or TAK1 siRNA, and 24 hours later with κB-Luc and pRL-TK control *Renilla* reporter at a ratio of 3∶1. The following day, cells were serum-starved overnight and treated with ligand (FGF1) for 8 hours prior to lysis and dual-luciferase assay. (**D**) MGHU3 cells were transfected with TAK1 or non-targeting siRNA for 48 hours, serum starved overnight then treated with FGF1 ligand for the time indicated. Cells were then fractionated, and 10 µg of nuclear fraction was run on an SDS-page gel and western blotted. Blots were probed with anti-p65 and anti-p84 (nuclear marker) antibodies. Densitometry was performed and p65 measurements were normalized to p84 measurements. (**E**) FGFR3 signaling is not altered by TAK1 knockdown. KMS11 cells were transfected with control or TAK1 siRNA and, 24 hours later, treated with or without FGFR inhibitor, PD173074 for an additional 24 hours. Western blots were probed with p-ERK, total ERK and TAK1 antibodies. Statistical analysis was performed using a t-test; (*) p<0.05; (**) p<0.01; (***) p<0.001. Four independent experiments were performed.

Activation of NFκB contributes to MM pathogenesis, enhancing growth, survival, and metastasis (reviewed in [28]), and also promotes survival of bladder cancer cells [29], [30]. Based upon the potential importance of NFκB activity and gene expression profiling results that implicate NFκB signaling as a target for the FGFR3 and TAK1 interaction, we evaluated the combined contribution of FGFR3 and TAK1 to NFκB activity in cancer cells using an NFκB-luciferase reporter assay. As shown in initial assessment of MM lines (Figure 4A), expression of constitutively active FGFR3 mutants dramatically increased NFκB transcriptional activity. To determine whether TAK1 is required for NFκB activation by FGFR3, siRNA knockdown of TAK1 was evaluated in MM and UC lines that express endogenous FGFR3. In all lines, whether expressing wild-type or mutant FGFR3, we observed significantly reduced NFκB activation following knockdown of TAK1 (Figures 4 B, C). Addition of ligand enhanced this effect in MGHU3 cells, likely by activating other FGF receptors [42]. As a final test of NFκB activation, nuclear localization of the active p65 subunit of NFκB was evaluated. MGHU3 cells were tested and showed an increase in nuclear p65 upon FGF1 ligand treatment, and levels of nuclear p65 were decreased upon TAK1 knockdown, which is consistent with the NFκB luciferase data (Figure 4D). Notably, TAK1 is not required for the major FGFR3-responsive MAPK signaling pathway, as evidenced by the inability of TAK1 knockdown to alter ERK phosphorylation by FGFR3 (Figure 4E). Taken together, these data suggest a novel signaling pathway in which FGFR3 activates NFκB via TAK1.

### Adhesive Properties of FGFR3 Positive Cancer Cell Lines Change in the Absence of TAK1

**Figure 5:**
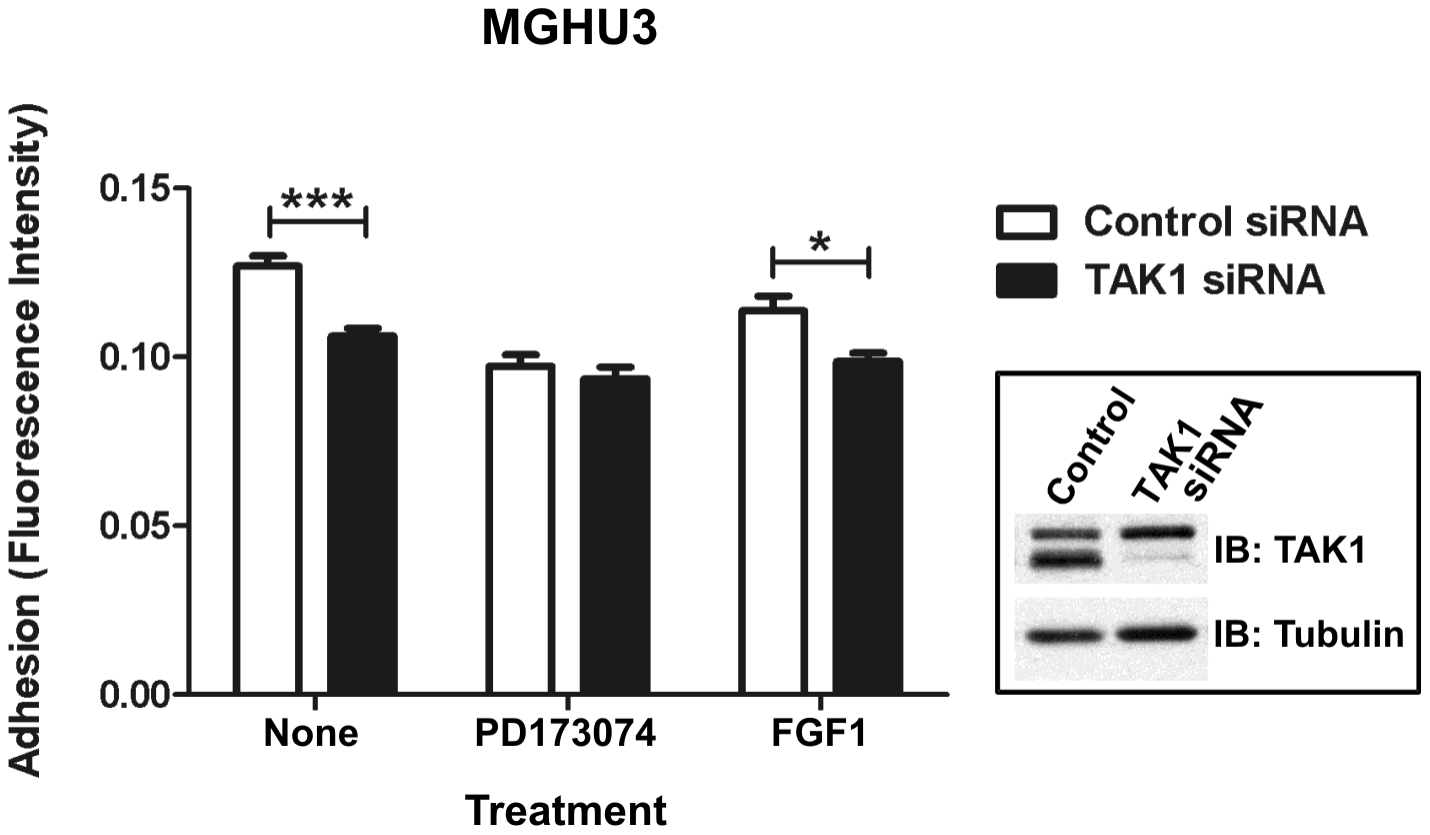
TAK1 knockdown reduces cell adhesion of FGFR3-associated bladder cancer. FGFR3-positive MGHU3 (Y375C) bladder cancer cells were transfected with control or TAK1 siRNA and treated with FGFR3 inhibitor (PD173074) or ligand (FGF1). PD173074 was added 3 hours after transfection and remained present through the duration of the experiment. FGF1-treated cells were serum-starved overnight and FGF1 added 1 hour prior to plating on collagen. Forty-eight hours post-transfection, cells were counted and plated on collagen type IV-coated tissue culture plastic. Cells were allowed to adhere 3 hours and adherence determined by fluorescence intensity following uptake of Calcein-AM. Statistical analysis was performed using a t-test; (*) p<0.05; (***) p<0.001; (****) p<0.0001. Representative of five experiments.

Cellular adhesion and migration are critical facets of cancer metastasis, in which altered adhesion to the extracellular matrix allows tumor cells to migrate away from the primary tumor to seed secondary sites [51]. NFκB induces expression of adhesion molecules, including ICAM-1, VCAM-1, and selectins (reviewed in [52]), and NFκB-dependent induction of VCAM-1 was recently reported to promote glioblastoma cell adhesion and invasion [53]. Several reports also define a role for TAK1 [25] and TAK1-NFκB [54], [55] signaling in the promotion of tumor cell adhesion, and FGFR3 mutations have been reported to decrease cellular attachment to extracellular matrix components in benign tumors [56]. As an initial assessment of the functional consequence of TAK1 and FGFR3 signaling, we evaluated cell adhesion in the MGHU3 UC line, based on previous studies using T24 bladder cancer cells [57]. In these cells, FGFR3 inhibition and TAK1 knockdown independently reduce cellular adhesion by approximately 20% (Figure 5). Importantly, simultaneous FGFR inhibition and TAK1 knockdown does not further decrease adhesion, consistent with FGFR3 signaling promoting cellular adhesion through TAK1.

## Discussion

This study describes the identification of a novel interaction between FGFR3 and TAK1, a member of the MAPK signaling pathway, both through molecular interaction and at the level of pathway integration. These pathways appear interdependent with selective functional effects on gene expression, NFκB activity and cell adhesion, all involved in oncogenesis. The role of FGFR3 in MM remains controversial, and a recent report implicates FGFR3 as an important driver of UC cell proliferation [42]. This novel interaction and selective effect on NFκB signaling provide new insights into these cancers with therapeutic implications.

The FGFR3-TAK1 interaction was initially identified by yeast two-hybrid screening and subsequently confirmed by Western and mass spectrometric analysis of co-immunoprecipitated proteins from multiple mammalian cell types, including FGFR3-associated malignancies (Figure 1, Table 1). We took a systems approach to examine the signaling integration that might be mediated by this novel FGFR3-TAK1 interaction. Gene expression profiling in UC cells identified 13 unique genes regulated by both TAK1 and FGFR3 (Table 2), which generate a single IPA network (Figure 3) with major hubs implicated in tumorigenesis, including NFκB. Tumor suppressor and other cancer-associated genes were also identified, including TNFAIP3 [58], SGK1 [59], and PSCA [60], which have been implicated in MM or bladder cancer. These findings may provide insight into common underyling mechanisms as well as identify candidates for future study and potential therapeutic development. It is intriguing that higher numbers of TAK1 specific gene changes were identified in the presence of FGFR3 inhibition compared to changes in the presence of active FGFR3. This could suggest that FGFR3 either has a complex impact on TAK1 signaling with both positive and negative effects, or that FGFR3 and TAK1 may also exert independent effects on other downstream pathways. The profiling results suggest that many genes that respond to input from both FGFR3 and TAK1 are associated with NFκB, notably in FGFR3-associated MM and UC. The result is consistent with previous studies indicating NFκB as highly activated and significant to MM pathogenesis [26], [61]. Less is known about the role of NFκB in bladder cancer; however, pathway inhibition can induce cell cycle arrest and inhibit proliferation [29], and NFκB nuclear expression is correlated with UC histological grade and T category [62].

We find that TAK1 can activate NFκB nuclear localization (p65) and transcriptional activity downstream of FGFR3 in both MM and bladder cancer cells (Figure 4 A-D). Furthermore, we confirm by qPCR, FGFR3-TAK1-mediated downregulation of TNFAIP3, a known NFκB target gene identified in our microarray analysis (Table 2; [63]). Other TAK1-regulated NFκB target genes identified by our microarray that have also been implicated in FGF signaling include BCL2L11, TNFAIP2, CCND1, CCL20 (MIP-3α), and BCL2L1 (Bcl-xL), the latter two shown to be regulated by FGF signaling in an NFκB-dependent manner [64]–[66]. The ability of FGFR3-TAK1 signaling to activate NFκB is interesting given that we map FGFR3 interaction with TAK1 to the same region (amino acids 441–579 of the C-terminal tail; Figure 1) as the TAB2/3 regulatory proteins (amino acids 479–547) required for TAK1 activation by Ser/Thr phosphorylation [50]. Further, both proteins interact constitutively with TAK1 (Figure 1 and [50]), raising the question of whether FGFR3 and TAB proteins bind TAK1 simultaneously, or whether separate pools of TAK1 with different binding partners exist. Given that TAK1 appears to be a substrate of FGFR3, both in culture and in cell-free kinase assay (Figure 2), it is possible that FGFR3 may activate TAK1 through a mechanism of tyrosine phosphorylation. Phosphotyrosine mapping functional analysis is in progress to address this question. This is the first published evidence of TAK1 tyrosine phosphorylation, although Netphos 2.0 server (http://www.cbs.dtu.dk/services/NetPhos/) has identified three to four tyrosine residues, depending on the TAK1 isoform, with high phosphorylation prediction scores. The importance of these data is further illustrated by the identification of novel FGF receptor-mediated tyrosine phosphorylation of IKKβ, which lies downstream of TAK1 [67], [68]. The IKKβ tyrosine phosphorylation, in conjunction with our TAK1 phosphorylation data leads us to propose that NFκB signaling may be a critical component of FGF receptor cellular activity and oncogenic potential.

NFκB activation in MGHU3 bladder cancer cells is further enhanced by addition of ligand (Figure 4C). This is in contrast to the KMS11 and LP1 MM lines (Figure 4B, C) which have FGFR3 overexpression and constitutive activation resulting from the t(4∶14) translocation [3]–[5], as well as elevated NFκB activity due to loss of function mutations in TRAF3, which may account for the dampened ligand responsiveness [26], [27]. Ligand responsiveness in MGHU3 cells may result from the stimulation of other FGF receptors, which we have not examined in detail, as FGFR3 is the most relevant FGF receptor for this cancer type. Recent studies demonstrate that FGFR3 and FGFR1 expression in UC cells are restricted to cells which also express epithelial or mesenchymal markers, respectively [42]. FGFR2 and FGFR4 exhibit a similar enrichment in epithelial or mesenchymal cells, respectively, but are expressed at much lower levels. These observations suggest that NFκB responsiveness to FGF1 in MGHU3 cells may be due to stimulation of FGFR2. While such a possibility would be surprising given recent reports that FGFR2 acts as a negative regulator of NFκB activity and suppresses tumor growth in UC cells [68], [69], it is consistent with our finding that FGFR2 can also interact with TAK1 (Figure 1E).

Importantly, silencing of TAK1 reduces NFκB activity to similar levels in the presence or absence of added ligand, suggesting that multiple FGF receptors may stimulate NFκB through interaction with TAK1. Indeed, we observe that FGFR1, 2, and 4 can all interact with TAK1 (Figure 1E), and overactivation of all has been associated with various human cancers, including those of the breast, lung, colon, endometrium, and prostate (reviewed in [10], [31]). It is therefore possible that TAK1-mediated activation of NFκB may be a common pathway of FGF receptor signaling and potentially relevant to multiple FGF receptor-associated malignancies.

Although FGFR3 can elicit effects on downstream signaling targets of TAK1, we found that TAK1 does not affect downstream FGFR3 signaling, as demonstrated by the inability of TAK1 knockdown to alter the ERK phosphorylation profile (Figure 4E and data not shown). This is in contrast to the effect of knocking down p85 subunits, which does modulate ERK1/2 phosphorylation [37]. These results suggest that the FGFR3/TAK1 effects are novel and distinct from the classically studied ERK/MAPK signaling pathways.

It was recently reported that TGFβ-Smad signaling promotes hepatic fibrosis and carcinogenesis in mice with a hepatocyte-specific deletion of TAK1 [70]. TGFβ is not likely to have the same effect here since TGFβ signaling is typically antagonized by FGF signaling through the ERK/MAPK pathway [71]–[73], and, in the case of MM cells, malignant cells express fewer surface receptors and are resistant to TGFβ signaling [74]–[77]. FGF signaling through ERK can phosphorylate Smad in some systems [78], [79], and TGFβ signaling can increase ERK/MAPK signaling by FGF receptors through downregulation of the negative regulator, Sprouty2 [80]; however, both function to inhibit Smad transcriptional activity, indicating that FGF is not likely to behave similarly to TGFβ in the absence of TAK1. However, this possibility was not evaluated in the current study.

Both FGFR3 mutations [56] and TAK1-NFκB signaling [53]–[55] have been implicated in the regulation of cell adhesion, alterations of which appear to have a central role in facilitating the metastatic process [51], [81]. Our initial evaluation of FGFR3 and TAK1 signals to adhesion of MGHU3 UC indicates that both function to promote cellular adhesion, possibly in a linear manner (Figure 5). These results are consistent both with the roles of FGFR3 and TAK1 in promoting cancer cell adhesion and invasion, and with previous studies which show reduced soft agar colony formation of MGHU3 cells following FGFR3 inhibition by siRNA or drug treatment [82]. However, FGFR1 and FGFR3 are reported to have largely non-overlapping roles in regulating invasion/metastasis or proliferation in UC cells expressing mesenchymal or epithelial markers, respectively [42]. Since we show that TAK1 can interact with FGF receptors 1–4 (Figure 1E), it will be important for future studies to consider the FGF receptor(s) expressed, as well as the cellular context.

Given the nearly identical signaling profiles in terms of ERK, NFκB and gene expression for this newly identified FGFR3-TAK1 pathway, it is likely that similar outcomes will be observed for the two cancers. However, unique outcomes are also possible given the different cell contexts, the tumor type, and the fact that FGFR3 mutations are associated with an early stage and less aggressive form of cancer in the bladder, while in MM, FGFR3 mutations are more associated with cancer progression (Reviewed in [2], [4]). Notably, the cellular context in which activating FGFR3 mutations are expressed is implicated in functional outcomes. In chondrocytes, activating FGFR3 mutations induce cell cycle arrest and premature senescence, but drive excessive proliferation in associated tumors, including multiple myeloma and bladder cancer ([83] and reviewed in [84]). These complex roles for FGFR3 in disease suggests that FGFR3 signaling outcomes may be related to cellular context and highlights the importance of systems wide approaches, such as described here, in understanding mechanisms and identifying therapeutic targets for disease specific treatments.

In this report, we provide evidence of a role for a highly integrated interaction between FGFR3 and TAK1 in bladder cancer and MM cases in which FGFR3 is implicated, laying the ground work for further understanding of these cancers and/or identification of other cancers in which these intersecting pathways are impacted. Finally, the specificity of gene expression modulation and impact on NFκB activation and other relevant oncogenic processes suggest the potential for highly selective therapeutic interventions.
